# Combined metabolome and transcriptome analysis revealed that MSTN regulated the process of bovine fatty acid metabolism in gut

**DOI:** 10.3389/fvets.2025.1541257

**Published:** 2025-04-28

**Authors:** Li Gao, Yong Ma, Lili Wang, Hao Wu, Ruobing Kang, Guangpeng Li, Lei Yang, Tong Wen

**Affiliations:** ^1^College of Ecology and Environment, Baotou Teacher’s College, Baotou, China; ^2^State Key Laboratory of Reproductive Regulation and Breeding of Grassland Livestock, College of Life Science, Inner Mongolia University, Hohhot, China

**Keywords:** MSTN, bovine, gut, metabolome, transcriptome

## Abstract

**Introduction:**

MSTN is a well-studied inhibitor of skeletal muscle development, but its mechanism of affecting gut metabolites and the functions it exerts through this pathway are still unclear. This study aims to reveal how MSTN affects the metabolism process by regulating gut metabolites.

**Methods:**

Combined analysis of jejunal contents metabolome and jejunal tissue transcriptome was used to compare the differences in intestinal metabolites and intestinal tissue gene expression between MSTN mutant and wild-type bovines.

**Results:**

Metabolomic analysis identified that compared to wild-type bovine, the abundance of 304 metabolites were significantly changed in MSTN mutant cattle including 142 upregulated and 162 downregulated. Transcriptome results showed that the expression level of 1541 genes were influenced by MSTN disruption, including 536 upregulated genes and 1005 downregulated genes, which were categorized into 311 KEGG signaling pathways, primarily related to disease and metabolism. Correlation analysis results suggested a notable cross-regulation between the transcript levels of some specific genes in jejunal tissues and the abundance of jejunal metabolites, represented by fatty metabolites and genes associated with fatty acid degradation, synthesis and elongation.

**Discussion:**

Collectively, the result of this study indicated that MSTN gene mutation led to alterations in gut microbial metabolites by increasing the abundance of beneficial monounsaturated fatty acids (MUFAs) such as oleic acid, then to promote fatty acid degradation while inhibiting its synthesis by regulating the expression levels of relevant genes. These results provide a foundation for understanding the effects of MSTN gene mutations on gut metabolites and its certain functions that MSTN regulated via gut metabolites

## Introduction

Muscle quality is one of the most important indicators in beef cattle breeding. Studies have shown that a member of transforming growth factor-β (TGF-β) known as myostatin (MSTN) or growth/differentiation factor 8, which mainly expressed in skeletal muscle, is essential in the negative regulation of muscle development (1). MSTN gene knockout in multiple species leads to the “double-muscle” trait due to increased numbers and sizes of muscle fibers, while MSTN overexpression inhibits muscle growth (2). Due to this property of the MSTN gene, researchers have begun to develop effective strategies to block MSTN gene expression to produce muscle-improved animals (3–7). Moreover, the MSTN gene is also widely involved in metabolic processes, and MSTN gene mutations can not only improve individual growth and mobility but also increase muscle yield and economic benefits in livestock production, making MSTN gene mutated animals significant in agricultural production.

Beyond the direct regulation of various metabolic pathways, recent studies have revealed that mutation of the MSTN gene can significantly affect the intestinal microecosystem of beef cattle (8, 9). The intestinal microbiota is abundant in the gastrointestinal tract and is considered the most important “microbial organ” within the animal body. It participates in the individual’s metabolic regulation, nutrient absorption, and immune function, playing a pivotal role in maintaining health and adaptive evolution of organisms (10, 11). Metabolite production and secretion is one of the ways in which gut microorganisms perform physiological functions on the host, the metabolites can function locally in the gut or enter into the bloodstream through the intestinal barrier, affecting the functions of multiple extra-intestinal organs (12). MSTN gene mutation has been reported to affect the gut microbiota in gene edited cattle (8, 9) and pigs (13), suggesting that MSTN mutation regulating host metabolism via gut microbes. However knowledge about its specific pathways is still limited, its role in gut microbiota and metabolites mediated metabolism remains unclear.

In the present study, the metabolic composition of intestinal contents of MSTN gene-edited and wild-type cattle were detected by LC-MS, then the gene expression in intestinal tissue of MSTN gene-edited and wild-type cattle were detected through transcriptomics, the combined metabolome and transcriptome analysis can help to understand the specific host metabolism pathways by which MSTN affected via gut metabolites.

## Materials and methods

### Ethics statement

This study was implemented in line with the recommendations for animal care and ethics in China. All animal experiments were allowed by the Animal Ethics and Welfare Committee of Baotou Teacher’s College (Approval Number: AEWC-BTTC2023001).

### Animals

The MSTN gene edited Luxi Yellow cattle used in this study were generated by CRISPR/Cas9 gene editing tools and somatic cell nuclear transfer as reported previously (8). Three 24-month-old wild-type female cattle and three female MSTN mutant cattle were chosen for sampling. The cattle were fed as common beef cattle in a local livestock farm.

### Sample collection

The cattle were fasted for 24 h before slaughter. The slaughter process complied with the national standard operating procedures (GB/T 19477-2018, Cattle Slaughtering, China). During the slaughter process, jejunal tissues and their contents were collected from both MSTN gene-edited and wild-type cattle, and samples were quickly placed in pre-cooled tubes, then immediately stored in liquid nitrogen and continuously preserved at −80°C for successive analysis.

### Metabolome analysis

Samples for metabolite determination were extracted from the jejunal contents of three wild-type and three MSTN gene mutant cattle. A high-resolution mass spectrometer (Waters Xevo G2-XS QTOF) controlled by MassLynx V4.2 was used to collect primary and secondary mass spectral data in MSe mode (Waters). A series of processing operations including peak extraction, peak alignment were carried out on raw data collected by MassLynx V4.2 using Progenesis QI software. Metabolites (theoretical fragment identification deviation and mass deviation ≥100 ppm) were charactered according to the information provide by METLIN online database and a self-constructed Biomark database in Progenesis QI software.

After normalizing the raw peak area information to total peak area, further analysis was carried out as follows. PCA and Spearman correlation analysis were conducted to assess the reproducibility of intra-group samples and quality control samples. Classification and pathway information for the metabolites were analyzed by KEGG database. Fold change of each group was calculated and then compared based on the grouping information, the significance of the content differences of each metabolite was obtained by t-test calculations. R package ropls was used to create the OPLS-DA model, and to verify its reliability, 200 times of permutation tests were conduct. The VIP (variable importance in projection) values were calculated by multiple cross-validation. All of the above parameters including fold change, *p*-value and VIP obtained from OPLS-DA model, together determine the metabolites that differ between groups. The selection criteria were set as: fold change (FC) >1.5, *p* < 0.05, and VIP >1. Hypergeometric distribution test was employed to calculate the significance of KEGG pathway enrichment for differential metabolites.

### Transcriptome sequencing

Total RNA was extracted from the jejunal tissues of three wild-type and three MSTN gene-mutated cattle using the RNAiso Plus kit (9108; Takara), with purity and concentration assessed using a NanoDrop 2000 spectrophotometer. After confirming sample quality, eukaryotic mRNA was enriched by magnetic beads modified with Oligo(dT) to construct libraries; the first and second cDNA strands were synthesized and then the cDNA was purified; the purified double-stranded cDNA underwent end repair, A-tailing, and ligation of sequencing adapters, and then performed size selection using AMPure XP beads; finally, conducted PCR enrichment to obtain the cDNA library. After quality control, Illumina NovaSeq6000 was used for the library sequencing (in PE150 mode). The clean data obtained after filtering is subsequently aligned with the specified genome sequence to obtain mapped data. FPKM (fragments per kilobase of transcript per million fragments mapped) value was used as an indicator to normalize the transcript or gene expression levels. Principal component analysis (PCA) was performed to assess the sample dispersion. The DESeq2 was used as a tool to determine differentially expressed genes (DEGs) according to the number of genes in each sample, with a cutoff of fold change ≥1.5 and false discovery rate (FDR) value <0.05 as screening criteria. All screened DEGs were subsequently mapped to the KEGG database.

### Real-time quantitative PCR

RNA from the tissue was isolated using an RNAiso Plus kit (9108; Takara), the RNA samples was subsequently reversed to cDNA by reverse transcription using a kit (R333-01; Vazyme). These cDNAs were used as templates for the next step, preparing a reaction system. In a 20 μL reaction system, the following components were mixed: cDNA, 8 μL; TB buffer, 10 μL; forward/reverse primer, 0.8/0.8 μL; sterile water, 0.4 μL. The reaction conditions were set as follows: initial denaturation (95°C for 30 s), 40 cycles of amplification (95°C for 10 s, 60°C for 30 s and 72°C for 30 s), with data collected during the elongation phase at 72°C. GADPH was served as the internal reference gene. qRT-PCR experiments were conducted, $2^{-\mathrm{\Delta \Delta CT}}$ method was used to calculate the differences in gene expression, with amplification conducted using a real-time PCR instrument. Primers used in this study were listed in Table 1.

**Table 1:** Primers used in the study.

Genes	Primers	Sequences (5′–3′)	Product size (bp)
MSTN	MSTN-F	CGCCTGGAAACAGCTCCTAA	181
	MSTN-R	ACTCCGTGGGCATGGTAATG	
ACACA	ACACA-F	CTGAACCAGCACTCCCGATT	240
	ACACA-R	CTTTCGGTCTCGACCTTGCT	
FASN	FASN-F	GCTGCAACTCAACGGGAACT	178
	FASN-R	ACCAGCTAGCACCACCTTCA	
ACOX3	ACOX3-F	CGTGGACTTTCTGGATGGCT	152
	ACOX3-R	CTCGACTGAGCTTCTGGTGG	
GAPDH	GAPDH-F	GTCGCCATCAATGACCCCTT	165
	GAPDH-R	GATGTTGGCAGGATCTCGCT	

### Joint analysis of metabolome and transcriptome

The joint analysis of the metabolome and transcriptome was conducted using two-way orthogonal PLS (O2PLS) and Pearson correlation to integrate and analyze the associations between the transcriptome and metabolome (platform BMKCloud^1^). A Venn diagram was applied to display the KEGG signaling pathways jointly enriched by the transcriptome and metabolome, while a bubble chart showed the significantly enriched signaling pathways (*p* ≤ 0.05).^2^ A Sankey diagram illustrated the associations between the signaling pathways, genes, and metabolites (see text footnote 2).

## Results

### Metabolite data analysis

**Figure 1:**
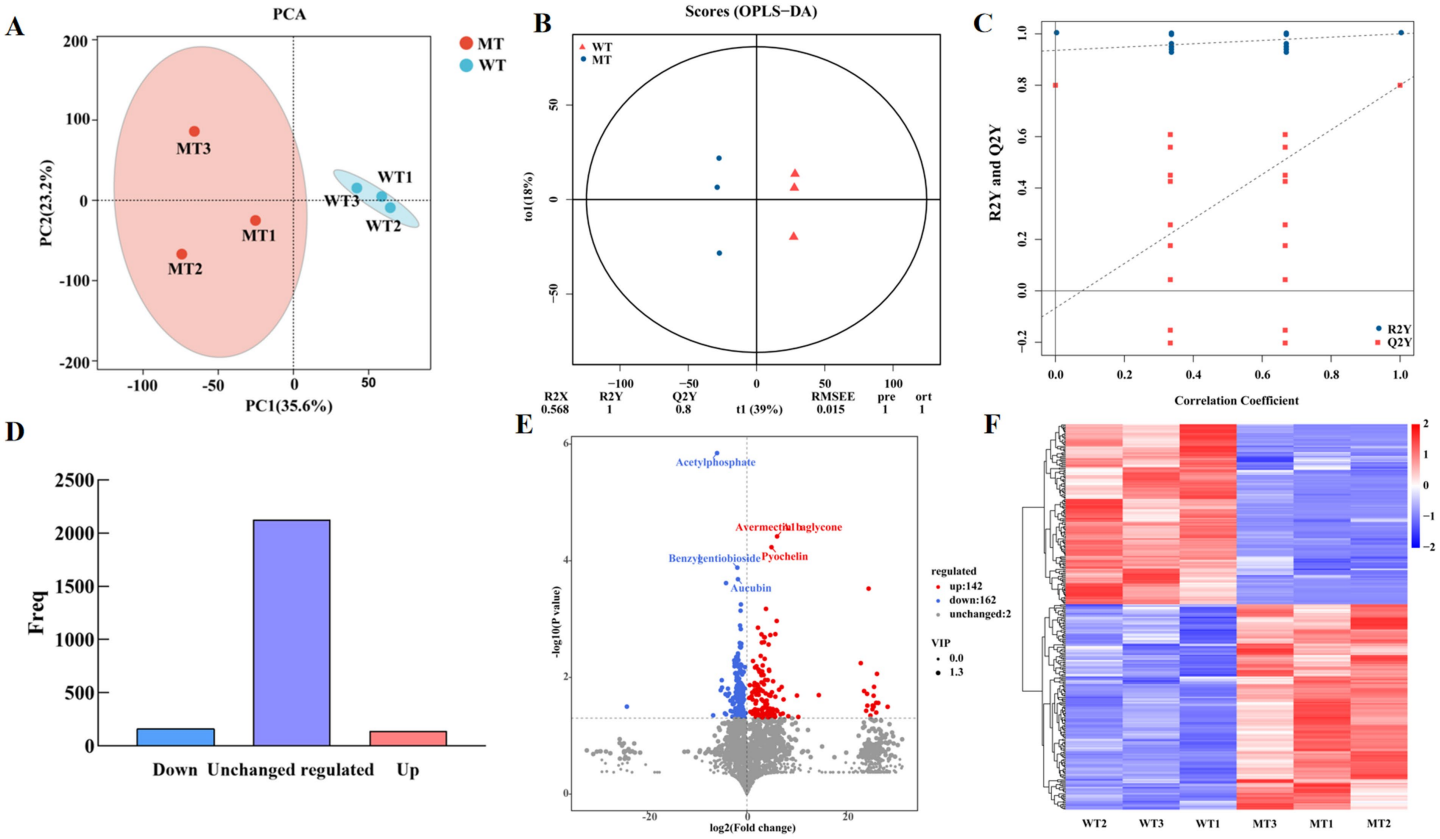
PCA and OPLS-DA of metabolites. **(A)** PCA score plot of metabolites. **(B)** OPLS-DA used for multivariate statistical analysis. **(C)** Permutation plot. The load range is −1 to 1. A load close to −1 or 1 indicates a strong influence of the variable on the component, while a load close to 0 indicates a weak influence. **(D)** Histogram of differential gene expression statistics. **(E)** Volcano plot of VIP and *p*-value screening. **(F)** Heatmap of different metabolites.

LC-MS/MS was used to analysis the metabolites in the jejunal contents of WT and MT cattle detection platform. PCA was conducted to analyze the reproducibility between samples, and the results were displayed in Figure 1A. The MSTN gene-edited group samples clustered together, while the wild-type group (WT) samples formed another cluster, indicating good reproducibility within the groups and significant differences between them. PC1 and PC2 represented 35.6 and 23.2% of the total variations (Figure 1A). An orthogonal partial least squares discriminant analysis (OPLS-DA) model was constructed to analyze the differential metabolites between the MT and WT groups, with permutation tests conducted on the OPLS-DA model, as displayed in Figures 1B,C. A significant trend of separation between the two groups. The OPLS-DA model parameters *R*^2^*Y* and *Q*^2^*Y* were 1 and 0.8, respectively, proving that the OPLS-DA model was effective. These results indicate that there are significantly different metabolites in the jejunal contents of MT and WT cattle.

Metabolites with FC ≥1 were selected, and those with VIP ≥1 were identified as differential metabolites. Totally, 2,432 metabolites were detected, among them, the abundance of 304 metabolites showed significant differences between MT and WT samples (Figures 1D,E), of which 142 were upregulated and 162 were downregulated. A hierarchical clustering heat map of the selected differential metabolites suggested significant differences between the two groups (Figure 1F).

### Analysis of differential metabolites

Differential metabolites were screened based on VIP values, and the top 10 differential metabolites in the MT and WT groups were listed in Supplementary Table S1, including acetylphosphate, 21,22-diprenylpaxilline, avermectin A1b aglycone, pyochelin, cholic acid, 11-dehydro-thromboxane B2, benzyl gentiobioside, N8,N′8-citryl-bis(spermidine), aucubin, and cobalt-precorrin 7. These metabolites are primarily involved in signaling pathways such as biosynthesis of metabolites (ko01110), biosynthesis of 12-, 14- and 16-membered macrolides (ko00522), biosynthesis of siderophore group nonribosomal peptides (ko01053), secondary bile acid biosynthesis (ko00121), arachidonic acid metabolism (ko00590), porphyrin metabolism (ko00860), and biosynthesis of cofactors (ko01240).

**Figure 2:**
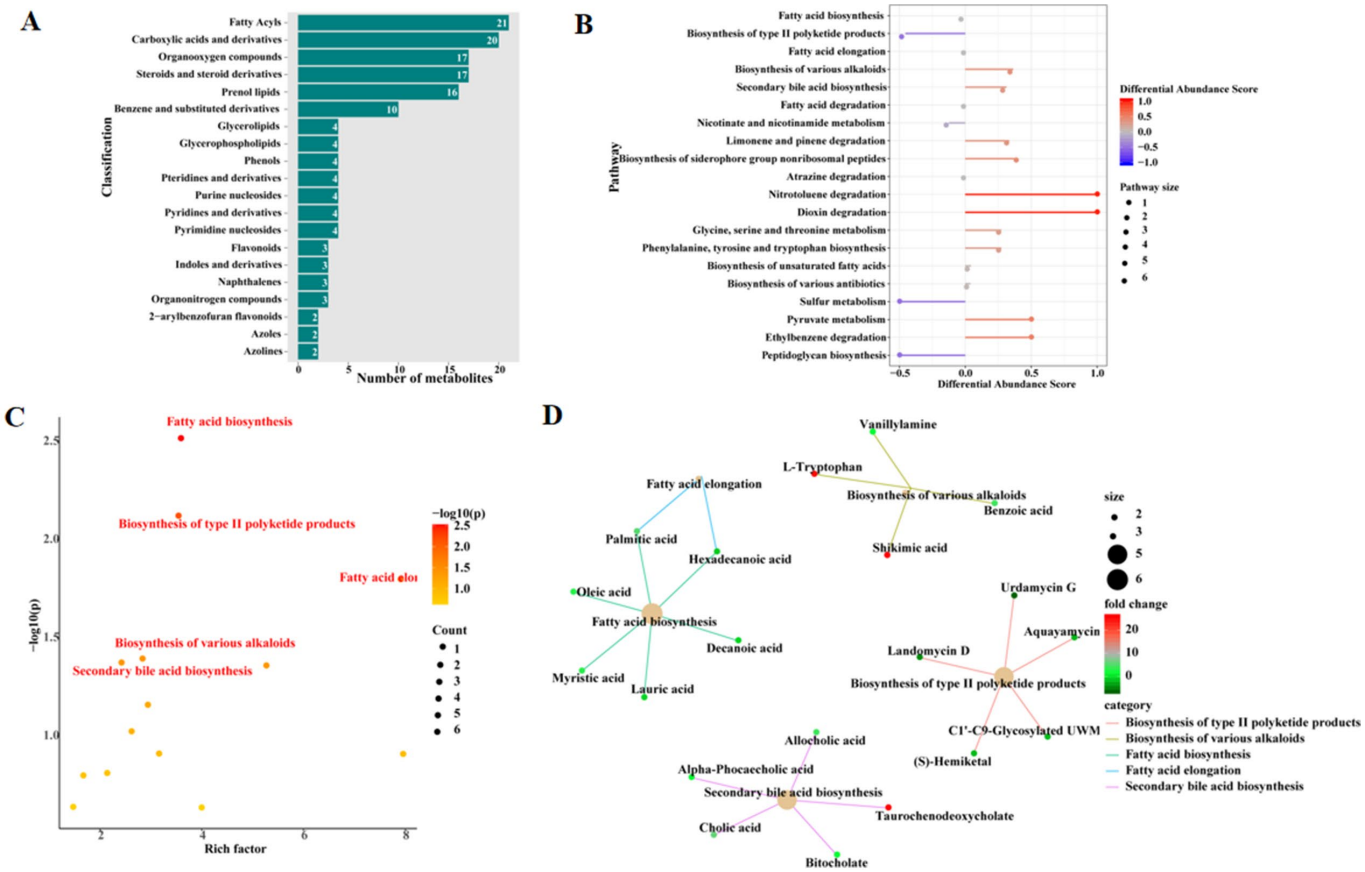
Differential metabolite analysis. **(A)** The top 20 differential metabolite classifications. **(B)** Top 20 KEGG pathways based on differential metabolite differential scores. **(C)** Pathway analysis based on the rich factor of metabolites. **(D)** KEGG pathway enrich cnetplot.

The metabolites with abundance differences in the top 20 in the MT and WT groups mainly include, carboxylic acids and derivatives, organooxygen compounds, steroids and steroid derivatives, prenol lipids, benzene and substituted derivatives, glycerolipids, glycerophospholipids, phenols, pteridines and derivatives, purine nucleosides, pyridines and derivatives, pyrimidine nucleosides, flavonoids, indoles and derivatives, naphthalenes, organonitrogen compounds, 2-arylbenzofuran flavonoids, azoles, and azolines (Figure 2A). Based on the differential metabolite scores, KEGG analysis was then performed. As was shown in Figure 2B. The KEGG-enriched metabolic pathways mainly included fatty acid biosynthesis, biosynthesis of type II polyketide products, fatty acid elongation, biosynthesis of various alkaloids, secondary bile acid biosynthesis, fatty acid degradation, nicotinate and nicotinamide metabolism, limonene and pinene degradation, atrazine degradation, biosynthesis of siderophore group nonribosomal peptides, dioxin degradation, nitrotoluene degradation, glycine, serine and threonine metabolism, phenylalanine, tyrosine and tryptophan biosynthesis, biosynthesis of unsaturated fatty acids, biosynthesis of various antibiotics, benzoate degradation, peptidoglycan biosynthesis, ether lipid metabolism, and pyruvate metabolism (Figure 2B).

The KEGG analysis of differential metabolites based on enrichment factors showed significant differences in the KEGG signaling pathways, including fatty acid biosynthesis, biosynthesis of type II polyketide products, fatty acid elongation, and biosynthesis of various alkaloids, as well as secondary bile acid biosynthesis (Figure 2C). The enrichment network diagram is shown in Figure 2D, where key differential metabolites in the fatty acid biosynthesis signaling pathway include lauric acid, decanoic acid, myristic acid, oleic acid, hexadecanoic acid, and palmitic acid. Key differential metabolites in the biosynthesis of type II polyketide products signaling pathway include urdamycin G, aquayamycin, landomycin D, C1′–C9-glycosylated UWM, and (S)-hemiketal. Key differential metabolites in the fatty acid elongation signaling pathway include palmitic acid and hexadecanoic acid. Key differential metabolites in the biosynthesis of various alkaloids signaling pathway include vanillylamine, L-tryptophan, benzoic acid, and shikimic acid. Key differential metabolites in the secondary bile acid biosynthesis signaling pathway include allocholic acid, alpha-phocaecholic acid, taurochenodeoxycholate, cholic acid, and bitocholate (Figure 2D).

### Quality assessment of transcriptome sequencing data

RNA-seq was used to compare the gene expression differences in the jejunal smooth muscle tissues of MSTN gene-edited cattle (MT1, MT2, and MT3) and wild-type cattle (WT1, WT2, and WT3). The average reads of per individual was 42,005,317, with a range from 38,858,456 to 47,116,854. The GC content of the data was mostly around 50%, and the Q30 values were all above 93%, indicating a high quality of the sequencing data (Table 2).

**Table 2:** Summary of transcriptome sequencing data.

Samples	Total reads	Mapped reads	Clean reads	Q30	GC content
MT1	41,366,154	38,531,858 (93.15%)	20,683,077	94.46%	48.73%
MT2	39,811,726	37,242,744 (93.55%)	19,905,863	93.45%	49.44%
MT3	47,116,854	44,900,056 (95.30%)	23,558,427	95.29%	49.45%
WT1	38,858,456	36,447,735 (93.80%)	19,429,228	93.66%	50.40%
WT2	45,127,958	41,981,785 (93.03%)	22,563,979	93.97%	50.51%
WT3	39,750,758	37,763,544 (95.00%)	19,875,379	94.58%	50.13%

### Analysis of differentially expressed genes

**Figure 3:**
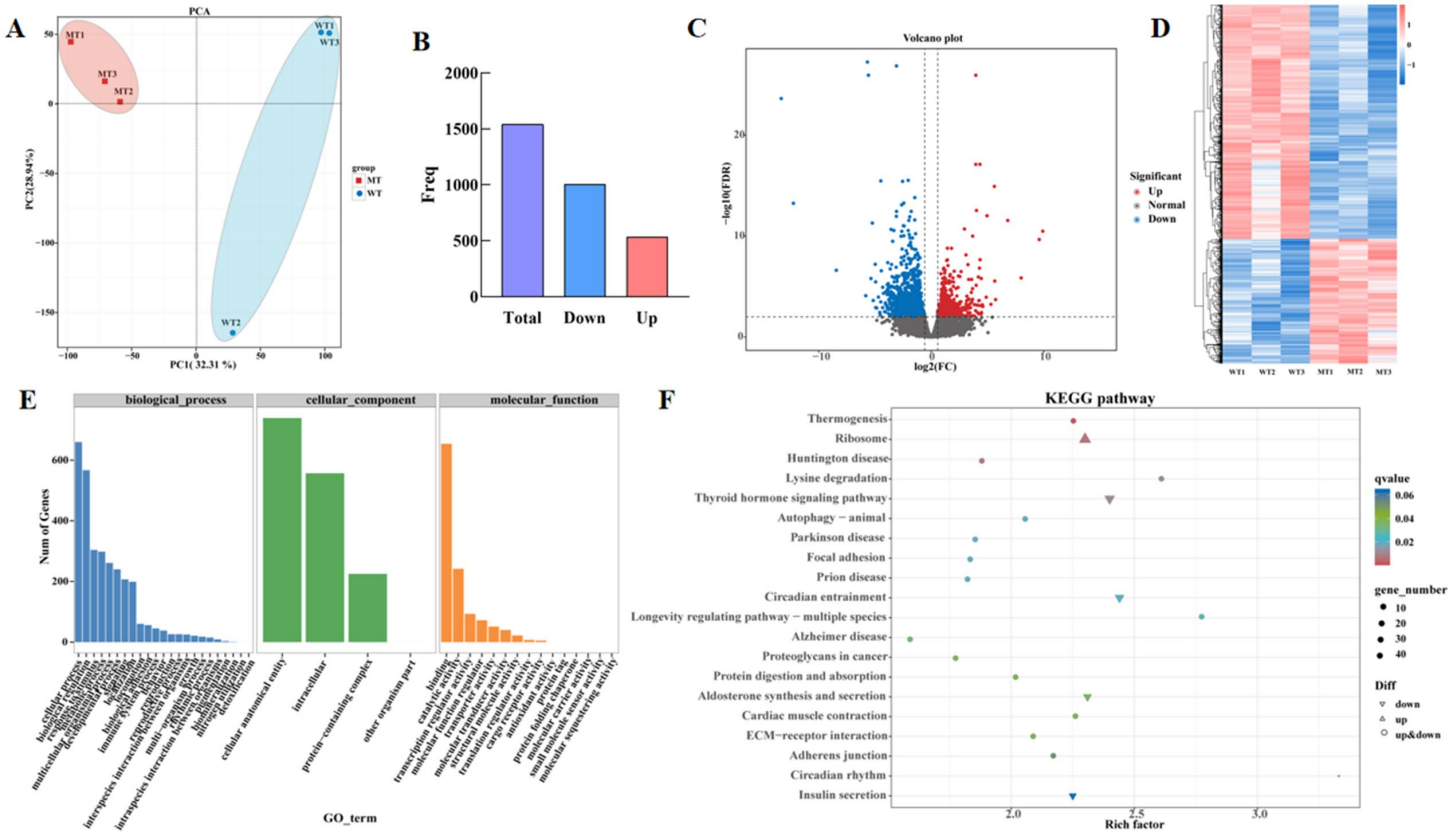
Differentially expressed genes in wild type cattle (WT) and MSTN mutant cattle (MT). **(A)** PCA score plot of transcriptomes. **(B)** Histogram of differential gene expression statistics. **(C)** Volcano plot of differentially expressed genes. **(D)** Subgroup clustering analysis. Red in the figure represents highly expressed genes, and blue represents low-expression protein-coding genes. **(E)** GO enrichment analysis, **(F)** KEGG enrichment analysis.

PCA was used to analyze the reproducibility between samples, the results were showed in Figure 1A. The samples from the MSTN gene-edited group clustered together, while the wild-type group (WT) samples formed another cluster, indicates that samples within groups are well reproducible, while significant differences are manifested between groups. 32.31 and 28.94% of the total variations were attributed to PC1 and PC2, respectively (Figure 3A). Based on gene read count data, differential expression calculations were performed with significant criteria set as FDR <0.05, FC ≥1.50 or ≤0.67. The results showed that a total of 1,541 genes were differentially expressed in the MT group compared with the WT group, of which 536 genes were up-regulated and 1,005 genes were down-regulated (Figures 3B,C). Hierarchical clustering results indicated significant differences in the expression profiles of genes between the two groups (Figure 3D). The top 10 differentially expressed genes were FNDC1, NCOR2, LOC540321, LOC539009, LOC617219, LOC404103, PTI, NCKAP5L, ARHGEF33, and CIC. The signaling pathways they participated in primarily included TGF-beta signaling pathway (ko04350); notch signaling pathway (ko04330); Epstein–Barr virus infection (ko05169); autophagy-animal (ko04140); mTOR signaling pathway (ko04150); renin-angiotensin system (ko04614); salivary secretion (ko04970); spinocerebellar ataxia (ko05017) (Supplementary Table S2).

GO enrichment was performed to identify significantly enriched DEGs, revealing that they were significantly enriched in 44 GO terms (*p* < 0.05). The significantly enriched biological processes mainly related to cellular processes, biological regulation, response to stimulus, and metabolic processes. The significantly enriched cellular components were primarily related to cellular anatomical entities, intracellular components, and protein-containing complexes. The significantly enriched molecular functions were mainly associated with binding, catalytic activity, translation regulator activity, molecular function regulators, and transporter activity (Figure 3E). Compared to the wild-type jejunal tissue transcriptome data, the MSTN gene-edited cattle enriched 311 KEGG signaling pathways, including thermogenesis, ribosome, Huntington disease, thyroid hormone signaling pathway, lysine degradation, circadian entrainment, prion disease, focal adhesion, Parkinson disease, autophagy-animal, longevity regulating pathway-multiple species, Alzheimer disease, proteoglycans in cancer, aldosterone synthesis and secretion, protein digestion and absorption, ECM-receptor interaction, cardiac muscle contraction, adherens junction, circadian rhythm, and insulin secretion (Figure 3F).

### Integrated analysis of DEGs and differential metabolites

**Figure 4:**
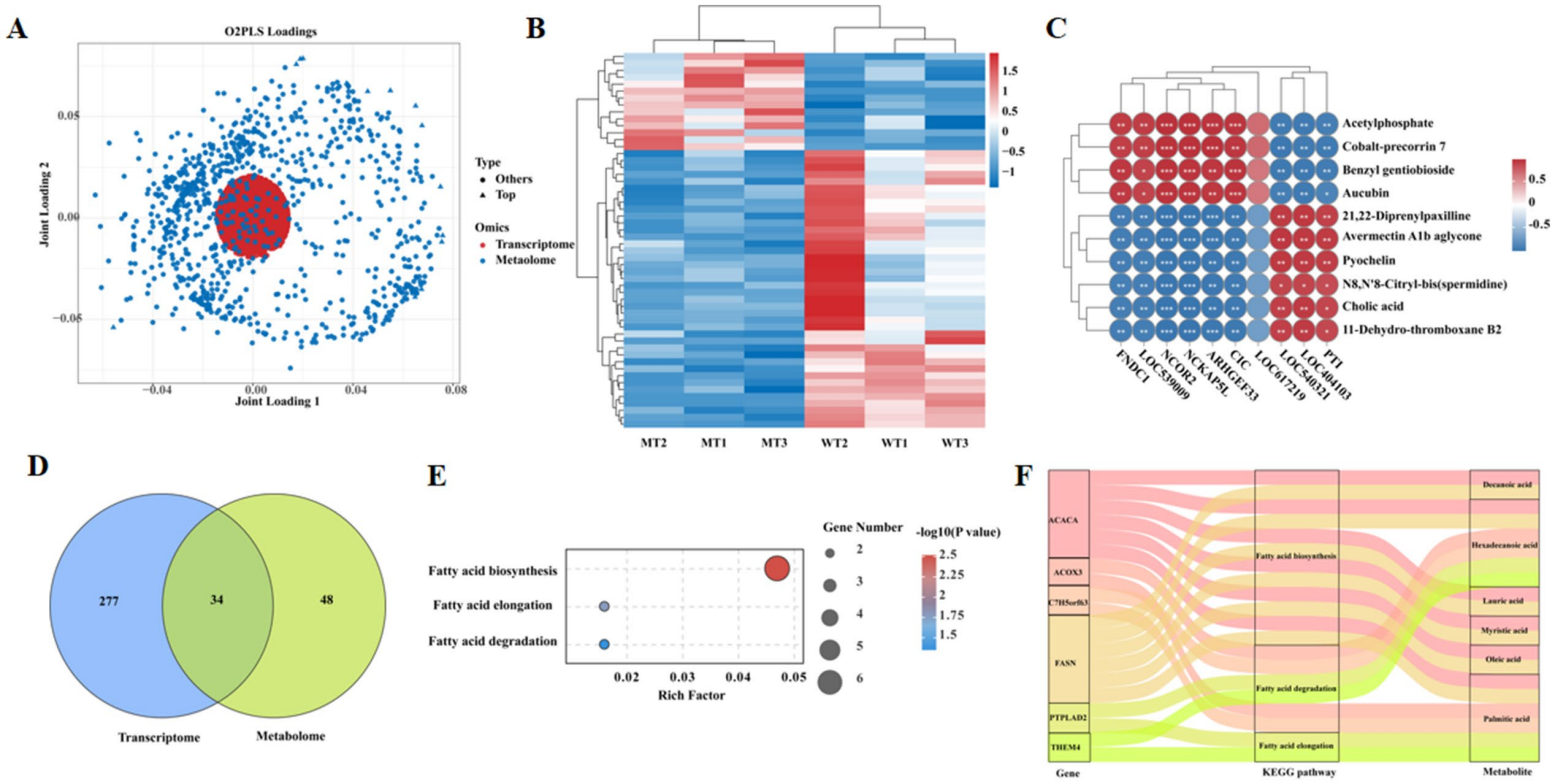
Joint metabolomic and transcriptomic correlation analysis. **(A)** O2PLS analysis. **(B)** Correlation heat map of transcripts and metabolites. **(C)** Correlation between top 10 differently expressed genes and top 10 differently metabolites. **(D)** Venn diagram of shared KEGG terms between transcriptome and metabolome. **(E)** Shared KEGG terms among transcriptome and metabolome. **(F)** Sankey of shared KEGG pathway.

The relationship between the transcriptome and metabolome was integrated using two-way orthogonal PLS (O2PLS), the results were displayed in Figure 4A, indicating significant associations between genes and metabolites. Correlation analysis and clustering of genes and metabolites from different samples revealed a significant clustering trend between the WT and MT group samples (Figure 4B). Pearson correlation analysis was conducted between the top 10 differential genes and the top 10 differential metabolites, revealing that FNDC1, LOC539009, NCOR2, NCKAP5L, ARHGEF33, and CIC were significantly positively correlated with acetylphosphate, cobalt-precorrin 7, benzyl gentiobioside, and aucubin; while they were significantly negatively correlated with 21,22-diprenylpaxilline, avermectin A1b aglycone, pyochelin, N8,N′8-citryl-bis(spermidine), cholic acid, and 11-dehydro-thromboxane B2. Conversely, LOC540321, PTI, and LOC404103 were significantly negatively correlated with acetylphosphate, cobalt-precorrin 7, benzyl gentiobioside, and aucubin; while they were significantly positively correlated with 21,22-diprenylpaxilline, avermectin A1b aglycone, pyochelin, N8,N′8-citryl-bis(spermidine), cholic acid, and 11-dehydro-thromboxane B2 (Figure 4C). Venn diagram analysis showed that there were 34 KEGG signaling pathways jointly enriched by the transcriptome and metabolome, with 277 KEGG signaling pathways unique to the transcriptome and 48 unique to the metabolome (Figure 4D). Among them, three pathways showed dramatic differences (*p* < 0.05) (Figure 4E), particularly pathways related to fatty acid metabolism including its biosynthesis, elongation and degradation. The differential genes and metabolites involved in these three signaling pathways were analyzed. Genes including ACACA and FASN which expressed differently between sample groups are involved in the fatty acid biosynthesis signaling pathway, while the main differential metabolites were hexadecanoic acid, oleic acid, lauric acid, myristic acid, decanoic acid, and palmitic acid. The differential genes involved in the fatty acid elongation signaling pathway included PTPLAD2 and THEM4, with the primary differential metabolite being palmitic acid. The differential genes involved in the fatty acid degradation signaling pathway included PTPLAD2, THEM4, ACOX3, and C7H5orf63, with the main differential metabolites being hexadecanoic acid and palmitic acid (Figure 4F).

**Figure 5:**
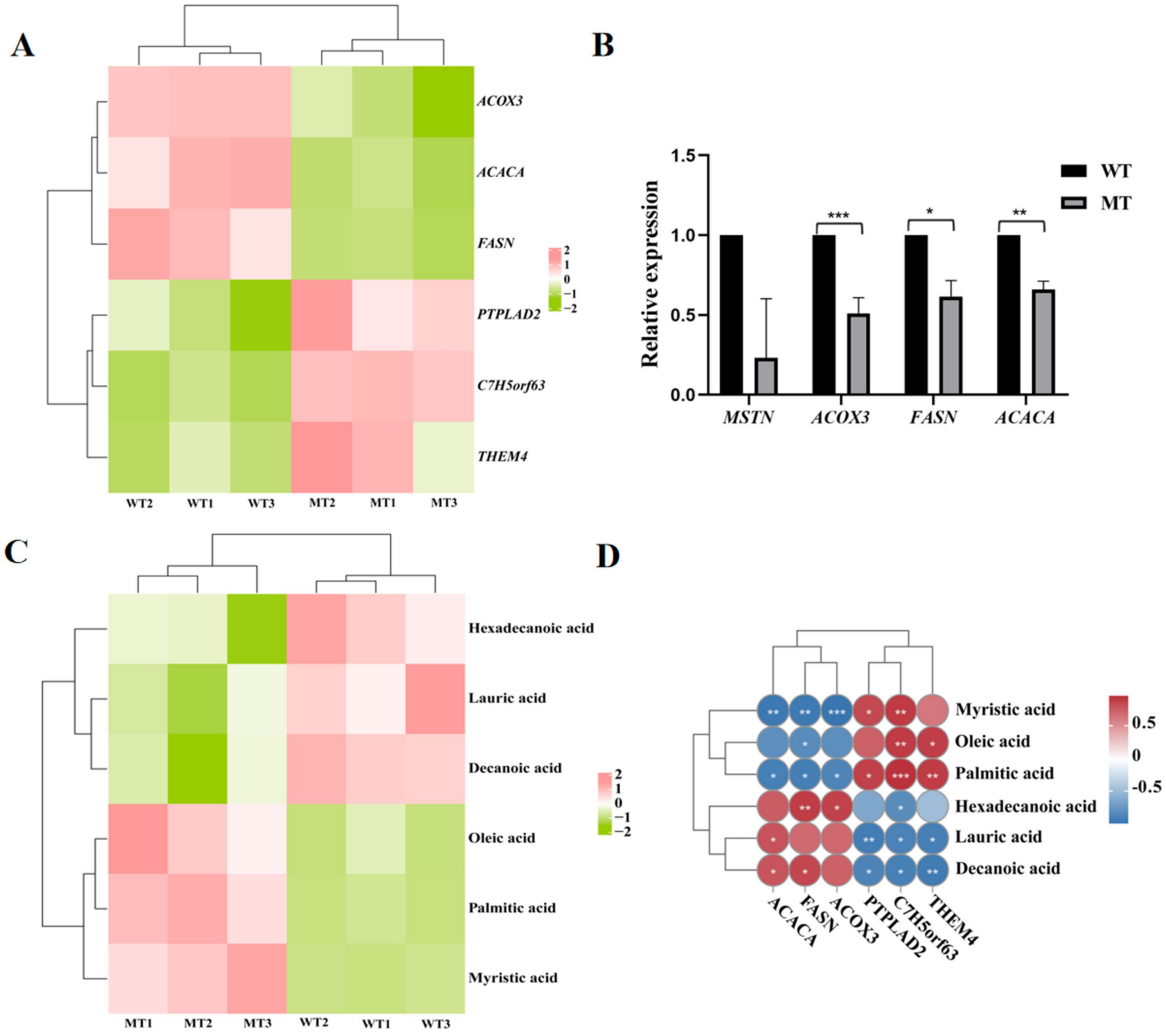
Correlation analysis of fatty acid metabolism-related genes and metabolites and their expression. **(A)** Heat map of fatty acid metabolism-related genes. **(B)** Real-time PCR validation of fatty acid metabolism gene expression. **(C)** Heat map of fatty acid metabolism-related metabolites. **(D)** Correlation analysis of fatty acid-related genes and metabolites. ^*^p < 0.05 and ^**^p < 0.01.

Analysis of the genes and metabolites involved in the three significantly different KEGG signaling pathways jointly enriched by the transcriptome and metabolome showed that ACOX3, ACACA, and FASN were significantly downregulated in the jejunal tissues of MSTN mutant cattle (Figure 5A), and this results were proved by real-time PCR (Figure 5B). PTPLAD2, C7H5orf63, and THEM4 were significantly upregulated in the jejunal tissues of MSTN mutant cattle (Figure 5A). Hexadecanoic acid, lauric acid, and decanoic acid were significantly downregulated in the jejunal tissues of MSTN gene mutant cattle, while oleic acid, palmitic acid, and myristic acid were significantly upregulated (Figure 5C). Correlation analysis of metabolites and genes involved in fatty acid metabolism showed that myristic acid was significantly negatively correlated with ACOX3, ACACA, and FASN, while it was significantly positively correlated with PTPLAD2 and C7H5orf63; oleic acid was significantly negatively correlated with FASN, while it was significantly positively correlated with C7H5orf63 and THEM4; palmitic acid was significantly negatively correlated with ACOX3, ACACA, and FASN, while it was significantly positively correlated with PTPLAD2, C7H5orf63, and THEM4; hexadecanoic acid was significantly positively correlated with FASN and ACOX3, while it was significantly negatively correlated with C7H5orf63; lauric acid was significantly positively correlated with ACACA, while it was significantly negatively correlated with PTPLAD2, C7H5orf63, and THEM4; decanoic acid was significantly positively correlated with ACACA and FASN, while it was significantly negatively correlated with PTPLAD2, C7H5orf63, and THEM4 (Figure 5D).

## Discussion

The yellow cattle is a unique beef cattle genetic resource in China with many distinctive features, however, the long period of artificial domestication has resulted in slow growth, low meat yield and underdeveloped hindquarters of this species, making it difficult to meet international beef standards. Therefore, technologies including molecular markers, genome-wide association analysis and gene editing have been utilized to explore and screen molecular markers for assessing the meat production performance of yellow cattle (14–17), thereby improving the accuracy and efficiency of yellow cattle breeding. As a widely studied regulator involved in the regulation of muscle development in animals, MSTN has also been shown to inhibit muscle development in cattle, and MSTN-deficient cattle can also develop a typical “double-muscle” phenotype. More intriguingly, MSTN deletion also leads to significant changes in the composition of the gut microbial community in cattle, but knowledge of how this change affects metabolism remains poor.

To reveal how MSTN mutations regulate host metabolic activity by affecting intestinal metabolites. in this study, we characterized the intestinal metabolome and jejunal tissue transcriptome of MSTN gene-edited and wild-type cattle as well as analyzed the association between them.

The analysis of metabolites in the intestinal contents showed that the most abundant categories of differential metabolites included fatty acyls, carboxylic acids and derivatives, organooxygen compounds, and steroids and steroid derivatives. Considering that the abundance of polyunsaturated fatty acid-producing microorganisms (Mortierella fungi), butyric acid-producing bacteria and some probiotic bacteria, was significantly higher in MSTN mutant cattle (8, 9), the altered composition of the microbial community is most likely responsible for the significant changes in metabolites represented by lipids and short-chain organic acids in the contents of the jejunum.

On the other hand, transcriptome analysis was used to identify the metabolic pathway enriched by genes whose expression level was significantly affected in intestinal tissue after MSTN mutation. The results revealed that the significantly affected KEGG pathways following MSTN gene mutation are involved in the biosynthesis, elongation and degradation of fatty acid. Previous studies have clarified that the MSTN inhibits muscle development while promoting fat synthesis. Study in mice proved that the β-oxidation of intramuscular fat in skeletal muscles was promoted and its synthesis was inhibited in MSTN mutants (18). Metabolite analyses of the liver revealed that MSTN gene mutations affect fatty acid metabolic pathways in cattle (19). Metabolite detection in pig adipose tissue also demonstrated that MSTN is involved in regulating fat metabolism, with reduced expression of MSTN ultimately lipid accumulation in subcutaneous fat was also affected (20). Proteomic studies of bovine gluteal muscles similarly showed that MSTN knockout increased the activity of several key enzymes in fatty acid β-oxidation and glycolysis (21). The current transcriptome data were consistent with the previous study that the mutation of MSTN can significantly affect the lipid metabolism pathway. Moreover, together with the significant changes in intestinal metabolites in MSTN mutants caused by the altered microbial community, correlation analyses were used to explore whether such changes leads to differential gene expression of related metabolic pathways.

In the gut of MSTN gene-mutated cattle, the level of an omega-9 fatty acid, oleic acid, was significantly upregulated. As a monounsaturated fatty acids (MUFA), oleic acid is well known for the function of decreasing cholesterol levels, reducing the risk of atherosclerosis (22–26), improving the host-graft response (27), controlling blood pressure and reducing the intake of daily antihypertensive medications (28). Additionally, oleic acid has anti-inflammatory properties and can alleviate autoimmune diseases (29, 30), and it also contributes to the prevention of breast cancer and the enhancement of immune function (31). The significant increase in beneficial oleic acid levels in the intestines of MSTN gene-mutant cattle suggested that the gut becomes healthier after MSTN gene mutation, consistent with previous findings that a remarkable increase in the abundance of probiotics in bovine intestinal was observed along with the deletion of MSTN (8). More than that, studies have shown that c15-20:1 FA including oleic acid, a C18:1 MUFA, can inhibit the expression of genes related to fatty acid synthesis in adipocytes and reduce triglyceride accumulation levels (32, 33), therefore to inhibit fatty acid synthesis and promote its degradation. This is consistent with the present transcriptome results that the significantly enriched metabolic pathways affected by MSTN gene mutation were related to fatty acid synthesis and metabolism. Specifically, fatty acid synthesis genes including ACACA and FASN were inhibited, while genes related to fatty acid elongation and degradation processes such as PTPLAD2 and THEM4 were promoted. This result also explained that the levels of saturated fatty acids such as hexadecanoic acid, lauric acid, and decanoic acid in metabolites were significantly reduced. Studies have shown that saturated fatty acids can increase cholesterol levels in the blood, triggering the onset of various cardiovascular diseases (34). Therefore, the decline of saturated fatty acid level will sure to have positive impact on health.

It was hypothesized based on the above results that in yellow cattle, the changes in gut microbiota caused by MSTN gene mutation led to a higher abundance of probiotics (8), and their metabolites including unsaturated fatty acid such as oleic acid promotes the degradation of saturated fatty acids by regulating the expression of genes related to fat metabolism in intestinal tissue. Eventually, through the accumulation of beneficial metabolites and the accelerated degradation of harmful metabolites, the synthesis and accumulation of fat is reduced and the health of the body is improved. Thus, MSTN editing or probiotic supplementation may demonstrate potential benefits for livestock breeding programs.

## Conclusion

In summary, the present study analyzed the metabolic composition of intestinal contents of MSTN gene-edited and wild-type cattle as well as the transcriptome of the jejunal tissues to elucidate the effects of MSTN gene mutations on gut metabolites and the certain pathways that MSTN regulated via gut metabolites. Results showed that the certain KEGG signaling pathways affected by MSTN gene mutation via gut metabolites were fatty acid biosynthesis, fatty acid elongation, and fatty acid degradation. Further analysis of the expression levels of related genes and metabolite abundances indicated that MSTN mutations improve host metabolic health by increasing beneficial fatty acids in the intestine (such as oleic acid), then they promoted the processes of fatty acid degradation, while inhibited the process of fatty acid synthesis. These results provide a foundation for understanding the effects of MSTN gene mutations on gut metabolites and its certain functions that MSTN regulated via gut metabolites.

## Data Availability

The datasets presented in this study can be found in online repositories. The names of the repository/repositories and accession number(s) can be found at: NCBI repository, accession number: PRJNA1149517, https://www.ncbi.nlm.nih.gov/bioproject/?term=PRJNA1149517.

## Footnotes

^1^ www.biocloud.net

^2^ https://www.omicstudio.cn/tool

## Glossary

MSTN: Myostatin
MUFA: Monounsaturated fatty acids
TGF-β: Transforming growth factor-β
LC-MS: Liquid chromatography mass spectrometry
OPLS-DA: Orthogonal partial least-squares discrimination analysis
KEGG: Kyoto Encyclopedia of Genes and Genomes
WT: Wild-type group
MT: MSTN mutant group
PCA: Principal component analysis
DEGs: Differentially expressed genes
qRT-PCR: Quantitative real-time PCR
O2PLS: Two-way orthogonal PLS
FC: Fold change
VIP: Variable importance in the projection
FDR: False discovery rate
GO: Gene Ontology
FNDC1: Fibronectin type III domain containing 1
NCOR2: Nucleus receptor corepressor 2
PTI: Pancreatic trypsin inhibitor
NCKAP5L: NCK-associated protein 5-like
PCR: Polymerase chain reaction
CIC: Capicua transcriptional repressor
ARHGEF33: Rhoguanine nucleotide exchange factor 33
ACACA: Acetyl-CoA carboxylase alpha
FASN: Fatty acid synthase
PTPLAD2: Protein tyrosine phosphatase-like domain containing 2
THEM4: Thioesterase superfamily member 4
ACOX3: Acyl-CoA oxidase 3
C7H5orf63: Chromosome 7 C5orf63 homolog
