# Supplementary material for: Combined metabolome and transcriptome analysis revealed that MSTN regulated the process of bovine fatty acid metabolism in gut
Source: Front Vet Sci. 2025 Apr 28;12:1541257. doi: 10.3389/fvets.2025.1541257 (PMC12066744; doi:10.3389/fvets.2025.1541257)
Supplement: Supplementary file 1 [file Table_1.docx]

Supplementary Material

## Supplementary Tables

**Table S1.** Top Ten Differential Metabolites.

| **Metabolites** | **log2FC** | **Pvalue** | **VIP** | **regulated** | **KEGG_annotation** | **KEGG_pathway_annotation** |
| --- | --- | --- | --- | --- | --- | --- |
| Acetylphosphate | -5.988923283 | 1.43E-06 | 1.602722762 | down | -- | -- |
| 21,22-Diprenylpaxilline | 24.478276 | 0.000300414 | 1.601607597 | up | C20561 | Biosynthesis of secondary metabolites(ko01110) |
| Avermectin A1b aglycone | 6.071600215 | 3.83E-05 | 1.601353002 | up | C11964 | Biosynthesis of 12-, 14- and 16-membered macrolides(ko00522);;Metabolic pathways(ko01100);;Biosynthesis of secondary metabolites(ko01110) |
| Pyochelin | 4.950879427 | 5.86E-05 | 1.601059029 | up | C12037 | Biosynthesis of siderophore group nonribosomal peptides(ko01053);;Biosynthesis of secondary metabolites(ko01110) |
| Cholic acid | 5.998408975 | 0.001076797 | 1.594196158 | up | C00695 | Secondary bile acid biosynthesis(ko00121);;Metabolic pathways(ko01100) |
| 11-Dehydro-thromboxane B2 | 5.731997697 | 0.001811932 | 1.592928487 | up | C05964 | Arachidonic acid metabolism(ko00590);;Metabolic pathways(ko01100) |
| Benzyl gentiobioside | -1.915471064 | 0.000131389 | 1.591460193 | down | -- | -- |
| N8,N'8-Citryl-bis(spermidine) | 22.89874237 | 0.005691974 | 1.586968393 | up | C22004 | Biosynthesis of siderophore group nonribosomal peptides(ko01053) |
| Aucubin | -1.793878531 | 0.000207403 | 1.584567519 | down | C09771 | -- |
| Cobalt-precorrin 7 | -4.188768014 | 0.000241527 | 1.583933596 | down | C16244 | Porphyrin metabolism(ko00860);;Metabolic pathways(ko01100);;Biosynthesis of cofactors(ko01240) |
