# Supplementary material for: Combined metabolome and transcriptome analysis revealed that MSTN regulated the process of bovine fatty acid metabolism in gut
Source: Front Vet Sci. 2025 Apr 28;12:1541257. doi: 10.3389/fvets.2025.1541257 (PMC12066744; doi:10.3389/fvets.2025.1541257)
Supplement: Supplementary file 2 [file Table_2.docx]

Supplementary Material

## Supplementary Tables

**Table S2.** Top 10 differential expressed genes.

| **Gene** | **FDR** | **log2FC** | **regulated** | **KEGG_pathway_annotation** | **NR_annotation** |
| --- | --- | --- | --- | --- | --- |
| FNDC1 | 5.39E-28 | -5.677500634 | down | TGF-beta signaling pathway (ko04350) | fibronectin type III domain-containing protein 1 isoform X1 [Bos taurus] |
| NCOR2 | 1.30E-27 | -3.092618362 | down | Notch signaling pathway (ko04330);; Epstein-Barr virus infection (ko05169) | TPA: nuclear receptor co-repressor 2-like [Bos taurus] |
| LOC540321 | 1.09E-26 | -5.588179277 | down | Autophagy - animal (ko04140);; mTOR signaling pathway (ko04150) | TPA: Rraga protein-like [Bos taurus] |
| LOC539009 | 1.09E-26 | 3.961445961 | up | Renin-angiotensin system (ko04614) | mast cell protease 2 [Bos taurus] |
| LOC617219 | 2.23E-24 | -13.33797824 | down | Salivary secretion (ko04970) | lysozyme C, tracheal isozyme isoform X1 [Bos taurus] |
| LOC404103 | 7.47E-18 | 4.329221295 | up | -- | TPA: spleen trypsin inhibitor I precursor [Bos taurus] |
| PTI | 7.51E-18 | 3.958638299 | up | -- | pancreatic trypsin inhibitor precursor [Bos taurus] |
| NCKAP5L | 2.89E-16 | -2.052290559 | down | -- | nck-associated protein 5-like isoform X2 [Bos taurus] |
| ARHGEF33 | 3.28E-16 | -4.486991467 | down | Autophagy - animal (ko04140);; mTOR signaling pathway (ko04150) | ras-related GTP-binding protein A [Bos taurus] |
| CIC | 3.63E-16 | -2.547886735 | down | Spinocerebellar ataxia (ko05017) | protein capicua homolog isoform X1 [Bos taurus] |
